# An Adaptive Cauchy Differential Evolution Algorithm for Global Numerical Optimization

**DOI:** 10.1155/2013/969734

**Published:** 2013-07-02

**Authors:** Tae Jong Choi, Chang Wook Ahn, Jinung An

**Affiliations:** ^1^Department of Computer Engineering, Sungkyunkwan University (SKKU), 2066 Seobu-ro, Suwon 440-746, Republic of Korea; ^2^Robot Research Division, Daegu Gyeongbuk Institute of Science and Technology (DGIST), 50-1 Sang-ri, Hyeonpung-meyeon, Daegu 711-873, Republic of Korea

## Abstract

Adaptation of control parameters, such as scaling factor (*F*), crossover rate (CR), and population size (NP), appropriately is one of the major problems of Differential Evolution (DE) literature. Well-designed adaptive or self-adaptive parameter control method can highly improve the performance of DE. Although there are many suggestions for adapting the control parameters, it is still a challenging task to properly adapt the control parameters for problem. In this paper, we present an adaptive parameter control DE algorithm. In the proposed algorithm, each individual has its own control parameters. The control parameters of each individual are adapted based on the average parameter value of successfully evolved individuals' parameter values by using the Cauchy distribution. Through this, the control parameters of each individual are assigned either near the average parameter value or far from that of the average parameter value which might be better parameter value for next generation. The experimental results show that the proposed algorithm is more robust than the standard DE algorithm and several state-of-the-art adaptive DE algorithms in solving various unimodal and multimodal problems.

## 1. Introduction

Differential Evolution (DE) is a powerful population based search technique for optimizing problems. Many researchers have used the DE in practical fields because this technique has good convergence properties and is easy to apply [1]. The DE has three control parameters such as scaling factor (*F*), crossover rate (CR), and population size (NP). The performance of DE is largely influenced by what parameter values are assigned to these control parameters. Therefore, in order to have a good optimization performance, finding suitable parameter values is of crucial importance [2, 3]. In the DE, the control parameters are usually adjusted by using the trial-and-error search method. However, the value assigned by the trial-and-error method might be efficient for solving one type of problems and inefficient for solving other problems [4]. Moreover, it requires a lot of computational resources. As a solution of this problem, parameter adaptation has been utilized. According to Eiben et al. [5, 6], the parameter adaptation can be categorized into three classes as follows.

1. Deterministic parameter control: the control parameters are adapted by some deterministic rule.
2. Adaptive parameter control: the control parameters are adapted by some form of feedback from evolutionary search.
3. Self-adaptive parameter control: the control parameters are adapted by the evolution-of-evolution technique. The control parameters are encapsulated in each individual as additional chromosomes and undergo evolutionary procedure.

The well designed adaptive or self-adaptive parameter control method can improve the performance of DE. Therefore, the adaptive and self-adaptive parameter controls are more applicable than the trial-and-error search method. So far, many adaptive and self-adaptive DE algorithms have been proposed and they have shown that the adaptive and self-adaptive DE algorithms have more robust performance than standard DE algorithm for many benchmark functions [4, 7, 8]. Although there are many suggestions for adapting control parameters, it is still a challenging task to properly adapt the control parameters for problem. Based on various experiments, we found out that the parameter adaptation should be performed in every generation and the control parameters of each individual should be adapted based on the average parameter value of successfully evolved individuals' parameter values by using the Cauchy distribution.

The Cauchy distribution is one of the long tail distributions. The Cauchy distribution generates large step from peak location with higher probability. Many evolutionary algorithms have used this long tail property as an escaping local minima method. The proposed algorithm also, but in different manner, utilizes the Cauchy distribution for the parameter adaptation. In the proposed algorithm, each individual has its own control parameters. The control parameters of each individual are adapted based on the average parameter value of successfully evolved individuals' parameter values by using the Cauchy distribution. It is because the successfully evolved individuals are led by appropriate parameter values. That is to say, the appropriate parameter values make the individuals take the good region for solving problem. However, there is a possibility that the current appropriate parameter values might be inappropriate parameter values in next generation. Therefore, we cannot assure that the parameter adaptation based on the average parameter value is good for making well-suited parameter values for future generations. In view of the above considerations, the parameter adaptation of proposed algorithm utilizes the Cauchy distribution as a large step method. According to it, the control parameters of each individual are assigned either near the average parameter value or far from that of the average parameter value which might be better parameter value for next generation. The experimental results show that the proposed algorithm is more robust than standard DE algorithm and some adaptive and self-adaptive DE algorithms such as jDE [4], SaDE [7], and MDE [9] on solving multimodal problems as well as unimodal problems.

The rest of this paper proceeds as follows. In Section 2, we introduce basic operations of standard DE algorithm and some adaptive and self-adaptive parameter control DE algorithms. In Section 3, the Cauchy distribution is described. The proposed algorithm is explained in detail in Section 4.  Section 5 presents the experimental results. We conclude this paper in Section 6.

## 2. Related Work

### 2.1. DE Algorithm

In the DE, a parent vector is called “target vector,” a mutant vector is that generated by mixing donor vectors, and an offspring obtained by making crossover between target vector and mutant vector is called “trial vector.” A target vector generates a trial vector which is moved around in search space by using the mutation and the crossover operations. If the fitness value of the trial vector is better than or equal to the fitness value of the target vector, the trial vector is accepted and included in the population of next generation, otherwise it is discarded and the target vector remains for the next generation. This cycle of operations is repeated until some specific termination conditions are not satisfied.

#### 2.1.1. Initialization

The population of the DE consists of NP individuals. Each individual is a *D*-dimensional parameter vectors, denoted as *X*_*i*,*G*_ = *x*_*i*,*G*_^1^,…, *x*_*i*,*G*_^*D*^ where *i* = 1,…, NP. In the initialization stage, first of all, the DE designates the search space of the test problem by prescribing the minimum (*X*_min⁡_ = *x*_min⁡_^1^,…, *x*_min⁡_^*D*^) and the maximum (*X*_max⁡_ = *x*_max⁡_^1^,…, *x*_max⁡_^*D*^) parameter bounds. After that, the parameter vectors of the each individual are initialized as follows:

(1) $$\begin{array}{c} x_{i,j,0}=x_{j,\text{MIN}}+\text{rand}\left[0,1\right]\cdot \left(x_{j,\text{MAX}}-x_{j,\text{MIN}}\right), \end{array}$$

where rand[0,1] is the uniform distributed random number lying between 0 and 1. By doing this, all the individuals are randomly scattered in the search space. After initialization, the DE executes a loop of the operations: mutation, crossover, and selection.

#### 2.1.2. Mutation Operation

The mutation is the first operation to generate child individuals from their parent individuals. So far, a lot of mutation strategies have been proposed. Here, we explain an example, called DE/rand/1/bin, which was introduced by Storn and Price [1]. First of all, the mutation strategy randomly select three mutually exclusive individuals among [1, NP]. They are called the “donor vectors”, denoted as *X*_*r*_1_,*G*_, *X*_*r*_2_,*G*_, and *X*_*r*_3_,*G*_. A mutant vector *V*_*i*,*G*_ is generated by adding the scaling difference of *X*_*r*_2_,*G*_ and *X*_*r*_3_,*G*_ to *X*_*r*_1_,*G*_.

(2) $$\begin{array}{c} V_{i,G}=X_{r_{1},G}+F\cdot \left(X_{r_{2},G}-X_{r_{3},G}\right), \end{array}$$

where *F* is a scaling factor for amplifying the difference value between *X*_*r*_2_,*G*_ and *X*_*r*_3_,*G*_.

#### 2.1.3. Crossover Operation

The crossover generates the trial vectors by making a crossover between target vector and mutant vector. There are two crossover operations which are commonly used. They are the binomial and the exponential crossovers. Here, we describe the binomial crossover. At the beginning, a random number is selected. If the random number is less than or equal to the crossover rate CR, the first element of the trial vector is occupied by the first element of the mutant vector. Otherwise, the element is occupied by the target vector. This procedure is repeated *D* times for each individual. The trial vector *U*_*i*,*G*_ = *u*_*i*,*G*_^1^,…, *u*_*i*,*G*_^*D*^ is generated as follows:

(3) $$\begin{array}{c} u_{i,j,G}=\{\begin{array}{cc} v_{i,j,G} & \text{if}\;\left(\text{rand}\left[0,1\right)\leq \text{CR}\;\text{or}\;j=j_{\text{rand}}\right)\\ x_{i,j,G\;} & \text{otherwise}. \end{array} \end{array}$$

Prior to crossover, the DE select another random number *j*_rand_ lying between [1, *D*]. The random number is used to guarantee that at least one element of the trial vector is occupied by the mutant vector.

#### 2.1.4. Selection Operation

The selection is the last operation of the DE iterations. It compares the fitness value of the target and the trial vectors. If the fitness value of the trial vector is better than or equal to the fitness value of the target vector, the trial vector is accepted and forms part of the population, otherwise it is discarded and the target vector remains for the next generation. these procedures are formulated as follows:

(4) $$\begin{array}{c} X_{i,G+1}=\{\begin{array}{cc} U_{i,G} & \text{if}\;\left(f\left(U_{i,G}\right)\leq f\left(X_{i,G}\right)\right),\\ X_{i,G} & \text{otherwise}. \end{array} \end{array}$$

### 2.2. jDE

Brest et al. [4] proposed a self-adaptive parameter control DE (called jDE) based on DE/rand/1/bin. In jDE, the control parameters, *F* and CR, are encapsulated in each individual as additional chromosomes. Therefore, all individuals have their own control parameters, denoted as *F*_*i*_ and CR_*i*_. jDE utilizes four additional parameters: *τ*_1_, *τ*_2_, *F*_*l*_, and *F*_*u*_. The first two parameters are used to determine whether the control parameters need to be updated or not and the last two parameters are used to designate the range of the control parameter *F*_*i*_. At the beginning, the values of *F*_*i*_ and CR_*i*_ are initialized by 0.5 and 0.9, respectively. Then, the control parameters *F*_*i*_ and CR_*i*_ for next generation are adapted as follows:

(5) $$\begin{array}{c} F_{i,G+1}=\{\begin{array}{cc} F_{l}+\text{ran}{\text{d}}_{2}\left[0,1\right]\cdot F_{u} & \text{if}\;\left(\text{ran}{\text{d}}_{1}\left[0,1\right]<\tau_{1}\right),\\ F_{i,G} & \text{otherwise}, \end{array}\\ \text{C}{\text{R}}_{i,G+1}=\{\begin{array}{cc} \text{ran}{\text{d}}_{4}\left[0,1\right] & \text{if}\;\left(\text{ran}{\text{d}}_{3}\left[0,1\right]<\tau_{2}\right),\\ \text{C}{\text{R}}_{i,G} & \text{otherwise}. \end{array} \end{array}$$

The author used *τ*_1_ = 0.1, *τ*_2_ = 0.1, *F*_*l*_ = 0.1, and *F*_*u*_ = 0.9. This procedure is executed before applying the mutation operation. Therefore, newly generated control parameters affect the mutation and the crossover operations.

### 2.3. DESAP

Teo [10] proposed the first self-adaptive population size DE (called DESAP) based on the self-adaptive Pareto DE [11]. DESAP can self-adapt not only the scaling factor *F* and the crossover rate CR but also the population size NP. This algorithm utilizes additional parameters such as *η*_*i*_, *δ*_*i*_, and *π*_*i*_. These parameters are encapsulated in each individual as additional chromosomes and also participated in the mutation and the crossover operations for evolving itself. The newly generated control parameters are selected when the fitness value of the trial vector is lower than or equal to the fitness value of the target vector. DESAP is divided into two algorithms (i.e., DESAP-abs and DESAP-rel) according to the equation of the population size for the next generation. DESAP has shown the effectiveness of the self-adaptive population size technique.

### 2.4. JADE

Zhang and Sanderson [12, 13] proposed a new mutation strategy called DE/current-to-*p*best which is lower greedy than DE/current-to-best/1. This strategy utilizes not the best individual of the population but the randomly selected one from the top 100*p*% (*p* ∈ (0,1]) individuals. In addition, an external archive scheme was proposed by storing the set of parameter vectors of recently discarded individuals. These parameter vectors provide the additional information about promising progress direction and increase the population diversity. The following equations represent the DE/current-to-*p*best with and without the external archive strategy:

(1) DE/current-to-*p*best with archive:
  (6) $$\begin{array}{c} V_{i,G}=X_{i,G}+F_{i}\cdot \left(X_{\text{best},G}^{p}-X_{i,G}\right)+F_{i}\cdot \left(X_{r1,G}-{\tilde{X}}_{r2,G}\right), \end{array}$$
(2) DE/current-to-*p*best without archive:
  (7) $$\begin{array}{c} V_{i,G}=X_{i,G}+F_{i}\cdot \left(X_{\text{best},G}^{p}-X_{i,G}\right)+F_{i}\cdot \left(X_{r1,G}-X_{r2,G}\right), \end{array}$$

where ${\tilde{X}}_{r2,G}$ is an individual randomly selected from the population or external archive.

In terms of parameter adaptation, JADE adapts the crossover rate CR_*i*_, as follows:

(8) $$\begin{array}{c} \text{C}{\text{R}}_{i}=\text{rnd}\;n_{i}\left(\mu_{\text{CR}},0.1\right), \end{array}$$

where rnd *n*_*i*_ is the Gaussian distributed random number generator. After that, the crossover rate CR_*i*_ is truncated to [0,1]. Moreover, *μ*_CR_ that is a mean value to generate CR_*i*_ is modified as follows:

(9) $$\begin{array}{c} \mu_{\text{CR}}=\left(1-c\right)\cdot \mu_{\text{CR}}+c\cdot {\text{mean}}_{A}\left(S_{\text{CR}}\right), \end{array}$$

where *c* is a constant value in [0,1], mean_*A*_ stands for the arithmetic mean, and *S*_CR_ contains the successfully evolved crossover rates of individuals after the selection operation. Similarly, the scaling factor *F*_*i*_ is adapted as follows:

(10) $$\begin{array}{c} F_{i}=\text{rnd}\;c_{i}\left(\mu_{F},0.1\right), \end{array}$$

where rnd *c*_*i*_ is the Cauchy distributed random number generator. After that, the scaling factor *F*_*i*_ is truncated to 1 if *F*_*i*_ ≥ 1 or regenerated if *F*_*i*_ ≤ 0. Also, *μ*_*F*_ that is a mean value to generate *F*_*i*_ is modified as follows:

(11) $$\begin{array}{c} \mu_{F}=\left(1-c\right)\cdot \mu_{F}+c\cdot \text{mea}{\text{n}}_{L}\left(S_{F}\right), \end{array}$$

where *c* is a constant value in [0,1], mean_*L*_ stands for the Lehmer mean, and *S*_*F*_ contains the successfully evolved scaling factors of individuals after the selection operation.

### 2.5. MDE

Ali and Pant [9] proposed a Modified Differential Evolution (MDE). This algorithm utilizes the Cauchy distribution as another mutation operation. In the selection operation, all individuals are monitored and the results of the selection operation are stored in the failure counter. If some individuals consequently fail to be selected as an individual for the next generation over MFC (Maximum Failure Counter), MDE assumes that these individuals were felled into some local minima. Therefore, the algorithm applies the Cauchy distributed mutation to these individuals instead of the mutation and the crossover operations to escape the local minima. After that, the failure counter is initialized by 0. MDE has shown the good performance for the higher dimensional problems, compared with DE/rand/1/bin.

## 3. Analysis of the Cauchy Distribution

The Cauchy distribution is a continuous probability distribution and it has two parameters *x*_0_ and *γ*. *x*_0_ is the peak location of the distribution and *γ* stands for the halfwidth at halfmaximum (HWHM) of the distribution. The value of *γ* determines the shape of the Cauchy distribution. If *γ* is assigned a lower value, the peak of the probability density function would be higher and its width would be narrower. On the other hand, if *γ* is assigned a higher value, the probability density function would have a lower peak and a wider width. The Cauchy distribution generates a large step from the peak with a higher probability. In general, many evolutionary algorithms have used this long tail property as an escaping local minima technique. The probability density function and the cumulative distribution function of the Cauchy distribution are defined by

(12) f(x;x0,γ)=1πγ[1+((x−x0)/γ)2]  =1π[γ(x−x0)2+γ2],F(x;x0,γ)=1πarctan((x−x0)/γ)+12.

**Figure 1:**
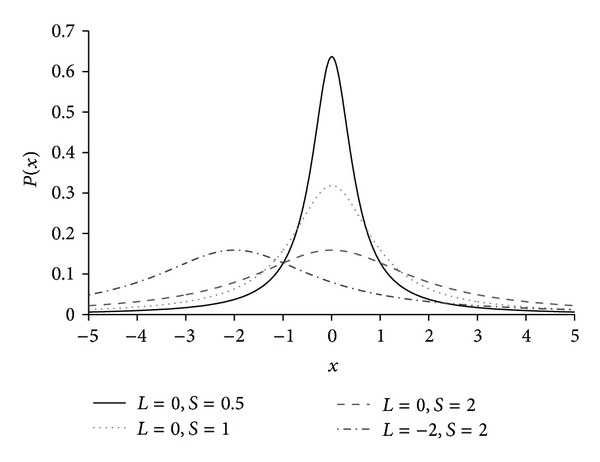
The various probability density functions of the Cauchy distribution.

Figure 1 illustrates the various probability density functions of the Cauchy distribution. Here, *L* and *S* denote the location (*x*_0_) and the scaling factor (*γ*), respectively. In addition, *L* = 0 and *S* = 1 generate the standard Cauchy distribution.

## 4. Adaptive Cauchy DE

### 4.1. When Parameter Adaptation Should Be Performed?

Finding appropriate moments of adapting control parameters is important problem for improving the DE performance. In this section, we explain when parameter adaptation should be performed.

Looking for previous studies, jDE utilizes self-adaptive method which allows each individual to maintain suitable control parameter values by itself. However, the parameter adaptation of jDE depends on the predefined probabilities (*τ*_1_ and *τ*_2_). Therefore, this method does not guarantee the adequacy of maintained control parameter values for current generation. In other words, it is possible that some individuals maintain unsuitable control parameter values. In SaDE, the scaling factor is calculated in every generation by using Gaussian distribution with the predefined mean value and all individuals utilize it. The crossover rate of SaDE, each individual has its own crossover rate and they are calculated by using Gaussian distribution with the median value of accumulated information about selection operation results during learning period as a mean value. This method is performed in every end of learning period. The parameter adaptation of SaDE has two problems. First, although each individual has different state during the DE iteration, the scaling factor adaptation of SaDE does not consider it. Therefore, many individuals might be utilized unsuitable control parameter values. Second, the selection operation results of past generations might become unnecessary or even noisy information for adapting the crossover rate. In addition, similar to jDE, during the learning period, it is possible that some individuals maintain unsuitable control parameter values.

Parameter adaptation should be performed whenever current control parameter values are not suitable for finding optimal value. We can utilize the selection operation results for distinguishing that an individual has suitable control parameters or not because the DE is based on the elitism. We find out that parameter adaptation should be performed in every generation. This means that every generation is appropriate moments of adapting control parameters. The reasons are follows.

1. If an individual has good control parameter values, the child individual can succeed in the selection operation and it can locate better region for finding optimal value than the region of its parent individual. At this moment, the characteristic of child individual region might differ from the characteristic of its parent region. It means that there is a possibility of the existing of more suitable control parameter values than the previous control parameter values for new region. Therefore, although an individual succeeds in the selection operation, we should apply parameter adaptation for finding more suitable control parameter values.
2. On the contrary, if an individual does not have good parameter values, the child individual might fail to evolve in the selection operation and then it remains the same region with its parent individual. This indicates that the individual needs more suitable control parameter values for escaping the region. Therefore, if an individual fails to evolve itself, we should also apply parameter adaptation.

As a result, because the individuals of DE are evolved for exploring new regions, it is hard to assure that the previous suitable control parameter values are still suitable until satisfying some probabilities or during some periods. Therefore, parameter adaptation should be performed in every generation.

### 4.2. How Parameter Adaptation Should Be Performed?

Finding proper method of adapting control parameters is also important problem for improving the DE performance. In this section, we explain how parameter adaptation should be performed. When performing parameter adaptation, we can utilize the successfully evolved individuals' control parameter values for parameter adaptation. It is because the successfully evolved individuals are led by good parameter values. That is to say, good parameter values make the individuals take the good region for solving problem.

Looking for previous studies, jDE adapts control parameter values by using the uniform distribution. However, the randomly generated control parameter values might not be suitable for finding better region. In SaDE, the successfully evolved individuals' crossover rates are stored in CR_Memory. After learning period, the parameter adaptation of SaDE extracts the median value from the CR_Memory as a mean value of the Gaussian distribution. In general, the median function is not largely influenced by outliers. However, the outliers give us the information about a new possibility of better control parameter values. Therefore, we should consider the outliers together.

As a result, the successfully evolved individuals' control parameter values based parameter adaptation is proper method to adapting control parameter values. This method should also consider the outliers.

### 4.3. The Proposed Algorithm

The proposed algorithm makes use of DE/rand/1/bin as a basic framework, in which the mutation is one of weaker greedy mutation strategies. In general, this mutation strategy is not so efficient in solving the unimodal problems since its lack of the fast convergence property makes the population slowly converge into the global minimum. However, if the control parameters are adapted suitably, this strategy can also demonstrate a good performance property in the unimodal and the multimodal problems.

The proposed algorithm adjusts two control parameters, *F* and CR, except for NP. The control parameter NP does not seriously affect the performance of DE more than the other two control parameters. Prior to explaining the adaptation procedures, the characteristics of these parameters are described. The control parameter *F* is related to the convergence speed of DE. Therefore, a higher value of *F* encourages the exploration power which is generally useful in the early stage of DE. On the other hand, a lower value of *F* promotes the exploitation power that is usually desirable in the later stage of DE. Moreover, the value of control parameter CR is related to the diversity of population.

The parameter adaptation of proposed algorithm utilizes *F*_Memory and CR_Memory. The successfully evolved individuals' scaling factors and crossover rates are stored in these memories. When performing parameter adaptation, arithmetic mean function is applied to extract mean values and these are actual parameter values of the Cauchy distribution as location parameters. The Cauchy distribution is one of the long tail distributions. The Cauchy distribution generates the large step from the peak location with higher probability. There is a possibility that the current appropriate parameter values might be the inappropriate parameter values in next generation. Therefore, we cannot assure that the average parameter value is still the well-suited parameter value for the future generations. In view of the above considerations, the parameter adaptation of the proposed algorithm utilizes the Cauchy distribution as a large step method. Through this, the control parameters of each individual are assigned either near the average parameter value or far from that of the average parameter value which might be the better parameter value of the next generation.

The details of the proposed algorithm are given as follows. First of all, all individuals have their own control parameters, *F*_*i*_ and CR_*i*_ where *i* is the individual's index. At the initialization stage, these parameters are initialized as 0.5 and 0.9, respectively. The mutation and crossover operations used in DE/rand/1/bin are employed. In the selection operation, if the trial vector is selected as an individual for the next generation, the control parameter values of this individual are stored in the *F*_Memory and CR_Memory. After the selection operation, the parameter adaptation is carried out.

The parameter *F*_*i*_ is adapted by the Cauchy distribution with the average parameter value. After that, the *F*_*i*_ is truncated to 0.1 or 1 if the *F*_*i*_ is less than 0.1 or greater than 1. The adaptation of the scaling factor is performed as follows:

(13) $$\begin{array}{c} F_{i,G+1}=C\left(0,\gamma_{F}\right)+F_{\text{avg},G}, \end{array}$$

where *F*_avg,*G*_ is the average parameter value of the accumulated information in the *F*_Memory as the location parameter of the Cauchy distribution. The *γ*_*F*_ is scaling factor of the equation and is assigned 0.1.

Similarly, the CR_*i*_ is adapted by the Cauchy distribution with the average parameter value. After that, the CR_*i*_ is truncated to 0 or 1 if the CR_*i*_ is less than 0 or greater than 1. The adaptation of the crossover rate is given as follows:

(14) $$\begin{array}{c} \text{C}{\text{R}}_{i,G+1}=C\left(0,\gamma_{\text{CR}}\right)+\text{C}{\text{R}}_{\text{avg},G}\text{,} \end{array}$$

where CR_avg,*G*_ is the average parameter value of the accumulated information in the CR_Memory as the location parameter of the Cauchy distribution. The *γ*_*F*_ is scaling factor of the equation and is assigned 0.1.  Algorithm 1 describes the pseudocode of the proposed algorithm.

When performing parameter adaptation, if there is no successfully evolved individual, then the average parameter values are assigned the average parameter values of last generation.

## 5. Performance Evaluation

### 5.1. Benchmark Functions

The performance of proposed algorithm was evaluated by fourteen benchmark functions. The first eleven benchmark functions are from [14, 15] and the rest benchmark functions are Extended *f*_12_ (*F*_12_), Bohachevsky (*F*_13_), and Schaffer (*F*_14_). The functions are shown in Table 1.

The characteristics of the benchmark functions are described as follows: *F*_1_–*F*_3_are continuous unimodal functions, *F*_4_ is a discontinuous step function, *F*_5_ is a noise quadratic function, and *F*_6_–*F*_14_ are continuous multimodal functions that the number of local minima exponentially increases when their dimension grows. A more detailed description of each function is given in [14, 15].

### 5.2. Experiment Setup

The proposed algorithm was compared to standard DE algorithm and several state-of-art adaptive DE algorithms. The five algorithms in comparison are listed as follows:

1. adaptive Cauchy DE;
2. DE/rand/1/bin with *F* = 0.5 and CR = 0.9 [1, 4, 16, 17];
3. jDE [4];
4. SaDE [7];
5. MDE with MFC = 5 [9].

All of the used parameter values are the recommended or utilized parameter values by their authors. The population size NP is fixed by 100 in all experiments. The maximum number of generations is assigned by 1500 for *F*_1_, *F*_4_, *F*_8_, *F*_10_, and *F*_11_; 2000 for *F*_2_ and *F*_9_; 3000 for *F*_5_, *F*_12_, and *F*_14_; 5000 for *F*_3_ and *F*_7_; 9000 for *F*_6_; 1000 for *F*_13_. All experiment results were run 50 times, independently. For clarity, the result of the best algorithm is marked in boldface. If the difference between the global minimum and the best fitness is lower than 10^−5^ (In *F*_5_, 10^−2^), we countered the experiment is successful.

### 5.3. Comparison of Adaptive Cauchy DE with Adaptive DE Algorithms

The mean and the standard deviation of experiment results obtained by adaptive Cauchy DE and the compared DE algorithms for *F*_1_–*F*_14_ for *D* = 30 are summarized in Table 2.

The proposed algorithm shows better performance on solving the unimodal problems as well as in the multimodal problems except *F*_3_ benchmark function. jDE shows the best performance in *F*_3_ benchmark function. The proposed algorithm outperformed all multimodal problems. The second best algorithm is jDE. Although SaDE utilizes strategy adaptation as well as parameter adaptation, jDE shows better results than SaDE in all benchmark functions. It means that parameter adaptation is more important to improve the performance of DE. MDE shows better performance than DE/rand/1/bin in several unimodal and multimodal problems.

Table 3 shows the success rate of comparison results. The success rate is obtained by a mount of successful counter divided by a mount of experiment runs (50). The proposed algorithm and two adaptive DE algorithms (jDE and SaDE) show perfect success rates. However, DE/rand/1/bin and MDE show lower success rate than the proposed algorithm and they totally failed to find global optimum in several benchmark functions.

**Figure 2:**
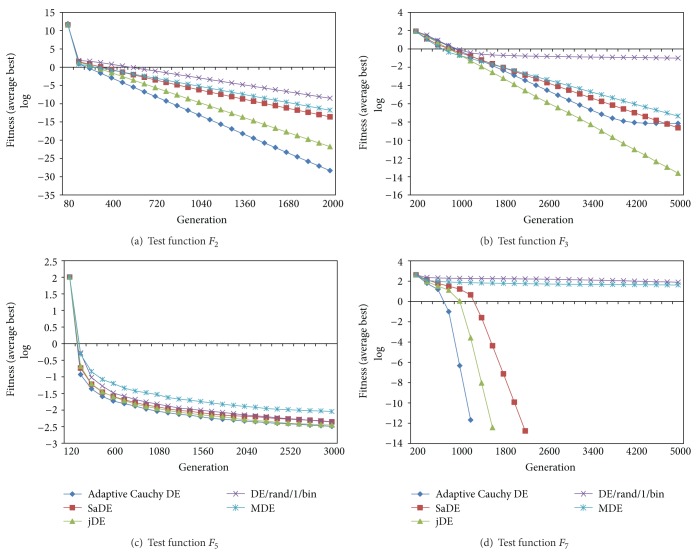
Average best graphs of Adaptive Cauchy DE with the compared DE algorithms.

Figure 2 shows the average best graphs of adaptive Cauchy DE and the compared DE algorithms.

### 5.4. Comparison of Adaptive Cauchy DE and FEP and CEP

The mean deviation and the standard deviation of experiment results obtained by adaptive Cauchy DE, DE/rand/1/bin, FEP (Fast Evolutionary Programming), and CEP (Classic Evolutionary Programming) for *F*_1_–*F*_11_ for *D* = 30 are summarized in Table 4. The results of FEP and CEP are taken from [13, Tables 2-4].

The proposed algorithm shows better performance on solving all benchmark functions than DE/rand/1/bin, FEP, and CEP. The second best algorithm is DE/rand/1/bin. However, DE/rand/1/bin shows lower performance than FEP in several benchmark functions (*F*_6_ and *F*_7_).

### 5.5. Comparison of Adaptive Cauchy DE and Adaptive LEP and Best Lévy

The mean deviation and the standard deviation of experiment results obtained by adaptive Cauchy DE, DE/rand/1/bin, adaptive LEP, and best Lévy for *F*_1_ and *F*_6_–*F*_11_ for *D* = 30 are summarized in Table 5. The results of adaptive LEP and best Lévy are taken from [18, Table 3]. The population size NP is fixed by 100 in all experiments. The maximum number of generations is assigned by 1500 for all benchmark functions.

The proposed algorithm shows better performance on solving all benchmark functions than DE/rand/1/bin, FEP, and CEP. The second best algorithm is DE/rand/1/bin again. However, DE/rand/1/bin shows lower performance than adaptive LEP and best Lévy in several benchmark functions (*F*_6_ and *F*_7_).

### 5.6. Parameter Study

Tables 6 and 7 show that various failure counter experiment results. The goal of this experiments is finding appropriate moments of parameter adaptation. FC_*F*_ = *n* means if an individual fails to evolve itself consequently *n* times, the scaling factor of individual is adapted. Similarly, FC_CR_ = *n* means if an individual fails to evolve itself consequently *n* times, the crossover rate of individual is adapted. For example, if FC_*F*_ = 1 and FC_CR_ = 0, then an individual's scaling factor is adapted when the individual fails to evolve itself, the last selection operation and the crossover of individual aer adapted in every generation.

The results show that adapting control parameters FC_*F*_ = 0 with FC_CR_ = 0 and FC_*F*_ = 1 with FC_CR_ = 0 had good performance in the comparison. However, when comparing success rate, FC_*F*_ = 0 with FC_CR_ = 0 had higher success rate than FC_*F*_ = 1 with FC_CR_ = 0 in *F*_3_ benchmark function.

Note that when failure counter is increasing, the performance of algorithm decreased. Therefore, parameter adaptation should be performed in every generation. This is because the individuals of DE are evolved for exploring new regions. Therefore, it is hard to assure that the previous suitable control parameter values are still suitable until satisfying some probabilities or during some periods.

Tables 8 and 9 show the various parameter adaptation method experiment results. The goal of these experiments is finding proper method of utilizing *F*_Memory and CR_Memory for parameter adaptation. Arithmetic mean indicates that the proposed algorithm utilized arithmetic mean function to extract mean values from *F*_Memory and CR_Memory and each individual's control parameter values are adapted based on the mean values as location parameters of the Cauchy distribution for parameter adaptation. Similarly, median indicates that the proposed algorithm utilized median function to extract median values from *F*_Memory and CR_Memory and each individual's control parameter values are adapted based on the median values as location parameters of the Cauchy distribution for parameter adaptation. Best individual indicates that each individual's control parameter values are adapted based on the best individual's control parameter values as location parameter of the Cauchy distribution. Finally, itself indicates that each individual's control parameter values are adapted based on its own control parameter values as location parameter of the Cauchy distribution.

The results show that adapting control parameters based on arithmetic mean function had good performance than median function. This is because the outliers give us the information about a new possibility of better control parameter values. Therefore, the arithmetic mean function is more applicable than median function. Parameter adaptation based on its own control parameter values shows good success rate in the comparison. However, the performance was lower than that of other methods. Parameter adaptation based on best individual's control parameter values shows good performance in only unimodal problems.

Tables 10 and 11 show the comparison results of the Cauchy distribution with the Gaussian distribution for parameter adapation method. The goal of these experiments is finding which distribution property (short or long tail) is more suitable for parameter adaptation. Cauchy *γ* = 0.1 indicates that parameter adaptation is performed based on the Cauchy distribution and the scaling parameter of distribution is assigned 0.1. Gaussian Std = 0.1 indicates that parameter adaptation is performed based on the Gaussian distribution and the standard deviation parameter of distribution is assigned 0.1.

The experiment results show that the Cauchy distribution with *γ* = 0.1 had good performance than others. This is because, the Cauchy distribution generates the large step from the peak location with higher probability. Therefore, the control parameters of each individual are assigned either near the average parameter value or far from that of the average parameter value which might be the better parameter value of the next generation.

## 6. Conclusion

The parameters of DE should be adequately assigned to attain better performance. But finding suitable values demands a lot of computational resources. In this sense, we present a new DE algorithm which utilizes success memories of scaling factors and crossover rates to properly adjust the control parameters of DE; the control parameters are adapted at each generation based on the Cauchy distribution with mean values of success memories. Experimental results showed that the adaptive Cauchy DE algorithm generally achieves better performance than existing DE variants on various multimodal and unimodal test problems. The results also supported the claim that a long tail distribution is more reliable than a short tail distribution in adjusting the control parameters.

## Figures and Tables

**Algorithm 1:**
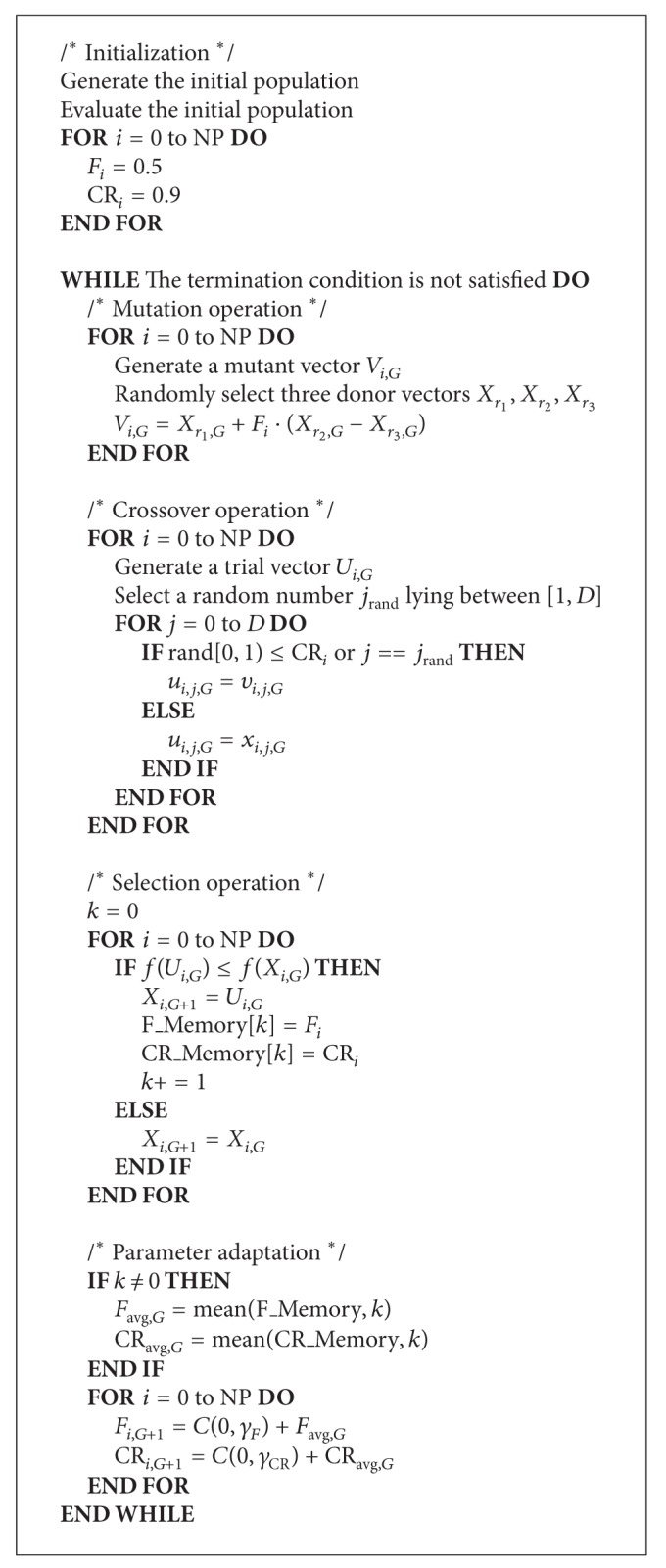
Adaptive Cauchy DE.

**Table 1:** Benchmark functions used in the performance evaluation.

Benchmark function	Dim	Search space	Global min.
$F_{1}(x)=\overset{D}{\underset{i=1}{\sum }}x_{i}^{2}$	30	[−100,100]^D^	0
$F_{2}(x)=\overset{D}{\underset{i=1}{\sum }}\left|x_{i}\right|+\prod_{i=1}^{D}\left|x_{i}\right|$	30	[−10,10]^D^	0
F_3_(x) = max⁡_i_⁡(|x_i_ | , 1 ≤ i ≤ D)	30	[−100,100]^D^	0
$F_{4}(x)=\overset{D}{\underset{i=1}{\sum }}{\left(\left\lfloor x_{i}+0.5\right\rfloor \right)}^{2}$	30	[−100,100]^D^	0
$F_{5}(x)=\overset{D}{\underset{i=1}{\sum }}ix_{i}^{4}+\text{random}\left[0,1\right)$	30	[−1.28,1.28]^D^	0
$F_{6}(x)=\overset{D}{\underset{i=1}{\sum }}-x_{i}\sin\left(\sqrt{\left|x_{i}\right|}\right)$	30	[−500,500]^D^	−12569.5
$F_{7}(x)=\overset{D}{\underset{i=1}{\sum }}\left(x_{i}^{2}-10\cos\left(2\pi x_{i}\right)+10\right)$	30	[−5.12,5.12]^D^	0
$F_{8}(x)=-20\exp\left(-0.2\sqrt{\frac{1}{D}\overset{D}{\underset{i=1}{\sum }}x_{i}^{2}}\right)-\exp\left(\frac{1}{D}\overset{D}{\underset{i=1}{\sum }}\cos2\pi x_{i}\right)+20+\exp\left(1\right)$	30	[−32,32]^D^	0
$F_{9}(x)=\frac{1}{4000}\overset{D}{\underset{i=1}{\sum }}x_{i}^{2}-\prod_{i=1}^{D}\cos\left(\frac{x_{i}}{\sqrt{i}}\right)+1$	30	[−600,600]^D^	0
$F_{10}\left(x\right)=\frac{\pi }{D}\left\{10\;\sin^{2}\left(\pi y_{1}\right)+\overset{D-1}{\underset{i=1}{\sum }}{\left(y_{i}-1\right)}^{2}\left[1+10\;\sin^{2}\left(\pi y_{i+1}\right)\right]+{\left(y_{D}-1\right)}^{2}\right\}\;$ $\;\;\;\;\;\;\;\;+\overset{D}{\underset{i=1}{\sum }}u\left(x_{i},10,100,4\right)$	30	[−50,50]^D^	0
$F_{11}\left(x\right)=0.1\left\{\sin^{2}\left(3\pi x_{1}\right)+\overset{D-1}{\underset{i=1}{\sum }}{\left(x_{i}-1\right)}^{2}\left[1+\sin^{2}\left(3\pi x_{i+1}\right)\right]+{\left(x_{D}-1\right)}^{2}\left[1+\sin^{2}\left(2\pi x_{D}\right)\right]\right\}\;$ $\;\;\;\;\;\;\;\;+\overset{D}{\underset{i=1}{\sum }}u\left(x_{i},5,100,4\right)$	30	[−50,50]^D^	0
$F_{12}(x)=f_{12}(x_{n},x_{1})+\overset{D-1}{\underset{i=1}{\sum }}f_{12}(x_{i},x_{i+1})$	30	[−100,100]^D^	0
f_12_(x, y) = (x^2^+y^2^)^0.25^[sin⁡^2^⁡(50(x^2^+y^2^)^0.1^) + 1]			
$F_{13}(x)=\overset{D-1}{\underset{i=1}{\sum }}\left(x_{i}^{2}+2x_{i+1}^{2}-0.3\cos\left(3\pi x_{i}\right)-0.4\cos\left(4\pi x_{i+1}\right)+0.7\right)$	30	[−15,15]^D^	0
$F_{14}(x)=\overset{D\;-\;1}{\underset{i=1}{\sum }}{\left(x_{i}^{2}+x_{i+1}^{2}\right)}^{0.25}\left[\sin^{2}\left(50{\left(x_{i}^{2}+x_{i+1}^{2}\right)}^{0.1}\right)+1\right]$	30	[−100,100]^D^	0

**Table 2:** The experiment result of comparison of adaptive Cauchy DE with other DE algorithms.

	GEN	Adaptive Cauchy DE		DE/rand/1/bin		jDE		SaDE		MDE	
		Mean	Std	Mean	Std	Mean	Std	Mean	Std	Mean	Std
F_1_	1500	5.0**E** − 36	9.4**E** − 36	7.9*E* − 14	6.8*E* − 14	2.6*E* − 28	4.0*E* − 28	1.8*E* − 20	2.3*E* − 20	7.0*E* − 17	2.8*E* − 17
F_2_	2000	2.4**E** − 30	1.6**E** − 30	1.2*E* − 09	6.7*E* − 10	1.8*E* − 23	1.8*E* − 23	6.2*E* − 15	3.2*E* − 15	4.8*E* − 13	1.3*E* − 13
F_3_	5000	2.9*E* − 12	1.1*E* − 12	3.3*E* − 02	7.2*E* − 02	2.0**E** − 15	3.3**E** − 15	7.8*E* − 10	2.0*E* − 10	2.0*E* − 08	8.5*E* − 09
F_4_	1500	0.0*E* + 00	0.0*E* + 00	0.0*E* + 00	0.0*E* + 00	0.0*E* + 00	0.0*E* + 00	0.0*E* + 00	0.0*E* + 00	0.0*E* + 00	0.0*E* + 00
F_5_	3000	3.0**E** − 03	6.9**E** − 04	4.6*E* − 03	1.0*E* − 03	3.1*E* − 03	8.5*E* − 04	4.5*E* − 03	1.2*E* − 03	8.8*E* − 03	1.8*E* − 03
F_6_	9000	− 12569.5	7.3**E** − 12	− 11095.3	5.2*E* + 02	− 12569.5	7.3**E** − 12	− 12569.5	7.3**E** − 12	− 11482.1	3.0*E* + 02
F_7_	5000	0.0**E** + 00	0.0**E** + 00	7.1*E* + 01	2.9*E* + 01	0.0**E** + 00	0.0**E** + 00	0.0**E** + 00	0.0**E** + 00	4.0*E* + 01	5.6*E* + 00
F_8_	1500	3.3**E** − 15	7.0**E** − 16	9.2*E* − 08	4.0*E* − 08	8.2*E* − 15	2.3*E* − 15	5.3*E* − 11	3.7*E* − 11	4.0*E* − 09	9.2*E* − 10
F_9_	2000	0.0**E** + 00	0.0**E** + 00	3.9*E* − 04	2.0*E* − 03	0.0**E** + 00	0.0**E** + 00	0.0**E** + 00	0.0**E** + 00	7.4*E* − 03	1.1*E* − 02
F_10_	1500	1.6**E** − 32	5.5**E** − 48	6.5*E* − 15	5.6*E* − 15	7.0*E* − 30	7.2*E* − 30	3.3*E* − 20	4.2*E* − 20	9.9*E* − 18	1.5*E* − 17
F_11_	1500	1.3**E** − 32	1.1**E** − 47	5.9*E* − 14	4.9*E* − 14	1.2*E* − 28	1.4*E* − 28	8.5*E* − 20	1.6*E* − 19	2.6*E* − 17	1.6*E* − 17
F_12_	3000	6.1**E** − 22	5.0**E** − 22	2.1*E* − 07	1.3*E* − 07	6.3*E* − 17	7.0*E* − 17	3.5*E* − 12	2.9*E* − 12	7.4*E* − 11	3.9*E* − 11
F_13_	1000	5.0**E** − 20	3.4**E** − 20	7.3*E* − 04	6.6*E* − 04	1.4*E* − 15	6.9*E* − 16	1.5*E* − 07	3.4*E* − 08	3.4*E* − 05	1.3*E* − 05
F_14_	3000	3.5**E** − 22	2.5**E** − 22	2.3*E* − 05	1.7*E* − 05	7.5*E* − 17	1.4*E* − 16	1.8*E* − 10	2.2*E* − 10	1.2*E* − 08	3.7*E* − 09

**Table 3:** The success rate of comparison of adaptive Cauchy DE with other DE algorithms.

Success rate	F_1_	F_2_	F_3_	F_4_	F_5_	F_6_	F_7_	F_8_	F_9_	F_10_	F_11_	F_12_	F_13_	F_14_
Adaptive Cauchy DE	100%	100%	100%	100%	100%	100%	100%	100%	100%	100%	100%	100%	100%	100%
DE/rand/1/bin	100%	100%	38%	100%	100%	0%	0%	100%	96%	100%	100%	100%	0%	18%
jDE	100%	100%	100%	100%	100%	100%	100%	100%	100%	100%	100%	100%	100%	100%
SaDE	100%	100%	100%	100%	100%	100%	100%	100%	100%	100%	100%	100%	100%	100%
MDE	100%	100%	100%	100%	72%	0%	0%	100%	58%	100%	100%	100%	0%	100%

**Table 4:** The experiment result of comparison of adaptive Cauchy DE with FEP and CEP.

	GEN	Adaptive Cauchy DE		DE/rand/1/bin		FEP		CEP	
		Mean	Std	Mean	Std	Mean	Std	Mean	Std
F_1_	1500	5.0**E** − 36	9.4**E** − 36	7.9*E* − 14	6.8*E* − 14	5.7*E* − 04	1.3*E* − 04	2.2*E* − 04	5.9*E* − 04
F_2_	2000	2.4**E** − 30	1.6**E** − 30	1.2*E* − 09	6.7*E* − 10	8.1*E* − 03	7.7*E* − 04	2.6*E* − 03	1.7*E* − 04
F_3_	5000	2.9**E** − 12	1.1**E** − 12	3.3*E* − 02	7.2*E* − 02	3.0*E* − 01	5.0*E* − 01	2.0*E* + 00	1.2*E* + 00
F_4_	1500	0.0**E** + 00	0.0**E** + 00	0.0**E** + 00	0.0**E** + 00	0.0**E** + 00	0.0**E** + 00	5.8*E* + 02	1.1*E* + 03
F_5_	3000	3.0**E** − 03	6.9**E** − 04	4.6*E* − 03	1.0*E* − 03	7.6*E* − 03	2.6*E* − 03	1.8*E* − 02	6.4*E* − 03
F_6_	9000	− 12569.5	7.3**E** − 12	− 11095.3	5.2*E* + 02	− 12554.5	5.3*E* + 01	− 7917.1	6.3*E* + 02
F_7_	5000	0.0**E** + 00	0.0**E** + 00	7.1*E* + 01	2.9*E* + 01	4.6*E* − 02	1.2*E* − 02	8.9*E* + 01	2.3*E* + 01
F_8_	1500	3.3**E** − 15	7.0**E** − 16	9.2*E* − 08	4.0*E* − 08	1.8*E* − 02	2.1*E* − 03	9.2*E* + 00	2.8*E* + 00
F_9_	2000	0.0**E** + 00	0.0**E** + 00	3.9*E* − 04	2.0*E* − 03	1.6*E* − 02	2.2*E* − 02	8.6*E* − 02	1.2*E* − 01
F_10_	1500	1.6**E** − 32	5.5**E** − 48	6.5*E* − 15	5.6*E* − 15	9.2*E* − 06	3.6*E* − 06	1.8*E* + 00	2.4*E* + 00
F_11_	1500	1.3**E** − 32	1.1**E** − 47	5.9*E* − 14	4.9*E* − 14	1.6*E* − 04	7.3*E* − 05	1.4*E* + 00	3.7*E* + 00

**Table 5:** The experiment result of comparison of adaptive Cauchy DE with adaptive LEP and best Lévy.

	GEN	Adaptive Cauchy DE		DE/rand/1/bin		Adaptive LEP		Best Lévy	
		Mean	Std	Mean	Std	Mean	Std	Mean	Std
F_1_	1500	4.7**E** − 36	5.1**E** − 36	7.2*E* − 14	7.9*E* − 14	6.3*E* − 04	7.6*E* − 05	6.6*E* − 04	6.4*E* − 05
F_6_	1500	− 12569.5	7.3**E** − 12	− 6506.71	6.7*E* + 02	− 11469.2	5.8*E* + 01	− 11898.2	5.2*E* + 01
F_7_	1500	0.0**E** + 00	0.0**E** + 00	1.7*E* + 02	1.2*E* + 01	5.9*E* + 00	2.1*E* + 00	1.3*E* + 01	2.3*E* + 00
F_8_	1500	3.2**E** − 15	5.0**E** − 16	9.1*E* − 08	3.7*E* − 08	1.9*E* − 02	1.0*E* − 03	3.1*E* − 02	2.0*E* − 03
F_9_	1500	0.0**E** + 00	0.0**E** + 00	2.2*E* − 13	1.4*E* − 13	2.4*E* − 02	2.8*E* − 02	1.8*E* − 02	1.7*E* − 02
F_10_	1500	1.6**E** − 32	5.5**E** − 48	7.5*E* − 15	7.2*E* − 15	6.0*E* − 06	1.0*E* − 06	3.0*E* − 05	4.0*E* − 06
F_11_	1500	1.3**E** − 32	1.1**E** − 47	5.4*E* − 14	4.9*E* − 14	9.8*E* − 05	1.2*E* − 05	2.6*E* − 04	3.0*E* − 05

**Table 6:** The experiment result of comparison of various failure counters.

	GEN	FC_F_ = 0, FC_CR_ = 0		FC_F_ = 0, FC_CR_ = 1		FC_F_ = 1, FC_CR_ = 0		FC_F_ = 1, FC_CR_ = 1		FC_F_ = 2, FC_CR_ = 2	
		Mean	Std	Mean	Std	Mean	Std	Mean	Std	Mean	Std
F_1_	1500	5.0*E* − 36	9.4*E* − 36	4.0*E* − 31	5.2*E* − 31	6.9**E** − 40	6.8**E** − 40	2.1*E* − 35	4.1*E* − 35	1.4*E* − 30	2.0*E* − 30
F_2_	2000	2.4*E* − 30	1.6*E* − 30	1.7*E* − 26	1.5*E* − 26	8.7**E** − 34	5.2**E** − 34	3.4*E* − 30	3.1*E* − 30	2.0*E* − 26	1.4*E* − 26
F_3_	5000	2.9**E** − 12	1.1**E** − 12	1.9*E* − 04	3.0*E* − 05	2.5*E* + 00	2.9*E* + 00	4.7*E* − 01	9.7*E* − 01	1.1*E* + 00	1.5*E* + 00
F_4_	1500	0.0*E* + 00	0.0*E* + 00	0.0*E* + 00	0.0*E* + 00	0.0*E* + 00	0.0*E* + 00	0.0*E* + 00	0.0*E* + 00	0.0*E* + 00	0.0*E* + 00
F_5_	3000	3.0**E** − 03	6.9**E** − 04	3.1*E* − 03	8.4*E* − 04	3.1*E* − 03	8.9*E* − 04	3.0*E* − 03	8.0*E* − 04	3.1*E* − 03	8.3*E* − 04
F_6_	9000	− 12569.5	7.3**E** − 12	− 12569.5	7.3**E** − 12	− 12569.5	7.3**E** − 12	− 12569.5	7.3**E** − 12	− 12567.1	1.7*E* + 01
F_7_	5000	0.0*E* + 00	0.0*E* + 00	0.0*E* + 00	0.0*E* + 00	0.0*E* + 00	0.0*E* + 00	0.0*E* + 00	0.0*E* + 00	0.0*E* + 00	0.0*E* + 00
F_8_	1500	3.3**E** − 15	7.0**E** − 16	1.4*E* − 14	4.5*E* − 15	3.1*E* − 15	0.0*E* + 00	3.0*E* − 07	2.1*E* − 06	1.5*E* − 14	2.6*E* − 15
F_9_	2000	0.0*E* + 00	0.0*E* + 00	0.0*E* + 00	0.0*E* + 00	0.0*E* + 00	0.0*E* + 00	0.0*E* + 00	0.0*E* + 00	0.0*E* + 00	0.0*E* + 00
F_10_	1500	1.6**E** − 32	5.5**E** − 48	9.6*E* − 32	6.6*E* − 32	4.8*E* − 07	3.4*E* − 06	1.6*E* − 32	3.6*E* − 34	1.7*E* − 31	1.6*E* − 31
F_11_	1500	1.3**E** − 32	1.1**E** − 47	7.3*E* − 31	1.1*E* − 30	1.4*E* − 32	6.9*E* − 34	1.3*E* − 32	1.1*E* − 47	8.5*E* − 31	7.8*E* − 31
F_12_	3000	6.1*E* − 22	5.0*E* − 22	1.6*E* − 18	1.8*E* − 18	1.4**E** − 23	6.9**E** − 23	2.0*E* − 21	2.1*E* − 21	1.4*E* − 18	1.4*E* − 18
F_13_	1000	5.0*E* − 20	3.4*E* − 20	1.7*E* − 16	1.4*E* − 16	5.8**E** − 23	3.3**E** − 23	8.6*E* − 20	5.9*E* − 20	4.7*E* − 17	4.1*E* − 17
F_14_	3000	3.5*E* − 22	2.5*E* − 22	7.2*E* − 19	7.1*E* − 19	9.5**E** − 25	1.1**E** − 24	8.0*E* − 22	8.9*E* − 22	8.3*E* − 19	6.6*E* − 19

**Table 7:** The success rate of comparison of various failure counters.

Success rate	F_1_	F_2_	F_3_	F_4_	F_5_	F_6_	F_7_	F_8_	F_9_	F_10_	F_11_	F_12_	F_13_	F_14_
FC_F_ = 0, FC_CR_ = 0	100%	100%	100%	100%	100%	100%	100%	100%	100%	100%	100%	100%	100%	100%
FC_F_ = 0, FC_CR_ = 1	100%	100%	0%	100%	100%	100%	100%	100%	100%	100%	100%	100%	100%	100%
FC_F_ = 1, FC_CR_ = 0	100%	100%	0%	100%	100%	100%	100%	100%	100%	98%	100%	100%	100%	100%
FC_F_ = 1, FC_CR_ = 1	100%	100%	0%	100%	100%	100%	100%	98%	100%	100%	100%	100%	100%	100%
FC_F_ = 2, FC_CR_ = 2	100%	100%	0%	100%	100%	98%	100%	100%	100%	100%	100%	100%	100%	100%

**Table 8:** The experiment result of comparison of various mathematical functions for utilizing success memories.

	GEN	Arithmetic mean		Median		Best individual		Itself	
		Mean	Std	Mean	Std	Mean	Std	Mean	Std
F_1_	1500	5.0**E** − 36	9.4**E** − 36	2.9*E* − 27	4.0*E* − 27	2.4*E* − 31	8.2*E* − 31	4.2*E* − 19	2.5*E* − 19
F_2_	2000	2.4**E** − 30	1.6**E** − 30	5.5*E* − 23	4.4*E* − 23	8.1*E* − 28	2.4*E* − 27	2.9*E* − 16	1.2*E* − 16
F_3_	5000	2.9**E** − 12	1.1**E** − 12	2.1*E* + 00	2.5*E* + 00	8.1*E* + 00	7.5*E* + 00	4.7*E* − 04	3.3*E* − 03
F_4_	1500	0.0*E* + 00	0.0*E* + 00	0.0*E* + 00	0.0*E* + 00	0.0*E* + 00	0.0*E* + 00	0.0*E* + 00	0.0*E* + 00
F_5_	3000	3.0**E** − 03	6.9**E** − 04	4.6*E* − 03	1.2*E* − 03	4.1*E* − 03	1.4*E* − 03	4.4*E* − 03	1.1*E* − 03
F_6_	9000	− 12569.5	7.3**E** − 12	− 12569.5	7.3**E** − 12	− 12569.5	7.3**E** − 12	− 12567.1	1.7*E* + 01
F_7_	5000	0.0*E* + 00	0.0*E* + 00	0.0*E* + 00	0.0*E* + 00	0.0*E* + 00	0.0*E* + 00	0.0*E* + 00	0.0*E* + 00
F_8_	1500	3.3**E** − 15	7.0**E** − 16	3.0*E* − 14	8.4*E* − 15	7.4*E* − 15	2.7*E* − 14	1.7*E* − 10	6.7*E* − 11
F_9_	2000	0.0**E** + 00	0.0**E** + 00	0.0*E* + 00	0.0*E* + 00	3.5*E* − 04	1.7*E* − 03	3.9*E* − 04	2.0*E* − 03
F_10_	1500	1.6**E** − 32	5.5**E** − 48	4.8*E* − 29	4.1*E* − 29	5.0*E* + 00	3.5*E* + 01	1.2*E* − 20	1.2*E* − 20
F_11_	1500	1.3**E** − 32	1.1**E** − 47	8.0*E* − 28	1.1*E* − 27	1.1*E* − 02	7.5*E* − 02	1.4*E* − 19	1.2*E* − 19
F_12_	3000	6.1**E** − 22	5.0**E** − 22	1.1*E* − 16	9.2*E* − 17	4.1*E* + 03	2.7*E* + 04	4.0*E* − 11	2.8*E* − 11
F_13_	1000	5.0**E** − 20	3.4**E** − 20	2.3*E* − 15	8.7*E* − 16	1.3*E* − 02	3.5*E* − 02	1.2*E* − 10	5.1*E* − 11
F_14_	3000	3.5**E** − 22	2.5**E** − 22	1.5*E* − 15	1.8*E* − 15	2.3*E* − 01	5.3*E* − 01	1.1*E* − 09	6.6*E* − 10

**Table 9:** The success rate of comparison of various mathematical functions for utilizing success memories.

Success rate	F_1_	F_2_	F_3_	F_4_	F_5_	F_6_	F_7_	F_8_	F_9_	F_10_	F_11_	F_12_	F_13_	F_14_
Arithmetic mean	100%	100%	100%	100%	100%	100%	100%	100%	100%	100%	100%	100%	100%	100%
Median	100%	100%	0%	100%	100%	100%	100%	100%	100%	100%	100%	100%	100%	100%
Best individual	100%	100%	0%	100%	100%	100%	100%	100%	96%	90%	96%	92%	74%	82%
Itself	100%	100%	90%	100%	100%	98%	100%	100%	96%	100%	100%	100%	100%	100%

**Table 10:** The experiment result of comparison of Cauchy distribution with Gaussian distribution for parameter adaptation.

	GEN	Cauchy γ = 0.1		Cauchy γ = 0.3		Gaussian Std = 0.1		Gaussian Std = 0.3	
		Mean	Std	Mean	Std	Mean	Std	Mean	Std
F_1_	1500	5.0**E** − 36	9.4**E** − 36	1.6*E* − 28	1.2*E* − 28	2.8*E* − 32	3.4*E* − 32	2.8*E* − 30	2.8*E* − 30
F_2_	2000	2.4**E** − 30	1.6**E** − 30	3.1*E* − 24	1.3*E* − 24	1.9*E* − 27	2.1*E* − 27	7.4*E* − 26	4.7*E* − 26
F_3_	5000	2.9**E** − 12	1.1**E** − 12	1.3*E* − 02	6.7*E* − 02	1.3*E* − 01	5.6*E* − 01	5.3*E* − 12	9.0*E* − 12
F_4_	1500	0.0*E* + 00	0.0*E* + 00	0.0*E* + 00	0.0*E* + 00	0.0*E* + 00	0.0*E* + 00	0.0*E* + 00	0.0*E* + 00
F_5_	3000	3.0**E** − 03	6.9**E** − 04	4.0*E* − 03	1.3*E* − 03	3.4*E* − 03	1.6*E* − 03	3.1*E* − 03	8.4*E* − 04
F_6_	9000	− 12569.5	7.3*E* − 12	− 12569.5	7.3*E* − 12	− 12569.5	7.3*E* − 12	− 12569.5	7.3*E* − 12
F_7_	5000	0.0*E* + 00	0.0*E* + 00	0.0*E* + 00	0.0*E* + 00	0.0*E* + 00	0.0*E* + 00	0.0*E* + 00	0.0*E* + 00
F_8_	1500	3.3**E** − 15	7.0**E** − 16	8.7*E* − 15	2.7*E* − 15	4.2*E* − 15	1.6*E* − 15	6.3*E* − 15	1.1*E* − 15
F_9_	2000	0.0**E** + 00	0.0**E** + 00	2.0*E* − 04	1.4*E* − 03	0.0**E** + 00	0.0**E** + 00	0.0**E** + 00	0.0**E** + 00
F_10_	1500	1.6**E** − 32	5.5**E** − 48	6.2*E* − 30	4.9*E* − 30	1.9*E* − 32	6.7*E* − 33	2.9*E* − 31	1.9*E* − 31
F_11_	1500	1.3**E** − 32	1.1**E** − 47	1.0*E* − 28	9.4*E* − 29	6.0*E* − 32	7.6*E* − 32	2.3*E* − 30	2.8*E* − 30
F_12_	3000	6.1**E** − 22	5.0**E** − 22	3.7*E* − 17	2.9*E* − 17	1.5*E* − 19	2.1*E* − 19	3.5*E* − 18	2.6*E* − 18
F_13_	1000	5.0**E** − 20	3.4**E** − 20	2.7*E* − 16	1.1*E* − 16	1.6*E* − 17	8.0*E* − 18	6.7*E* − 17	2.8*E* − 17
F_14_	3000	3.5**E** − 22	2.5**E** − 22	4.4*E* − 17	6.6*E* − 17	2.1*E* − 19	2.4*E* − 19	2.9*E* − 18	2.5*E* − 18

**Table 11:** The success rate of comparison Cauchy distribution with Gaussian distribution for parameter adaptation.

Success rate	F_1_	F_2_	F_3_	F_4_	F_5_	F_6_	F_7_	F_8_	F_9_	F_10_	F_11_	F_12_	F_13_	F_14_
Cauchy γ = 0.1	100%	100%	100%	100%	100%	100%	100%	100%	100%	100%	100%	100%	100%	100%
Cauchy γ = 0.3	100%	100%	78%	100%	100%	100%	100%	100%	98%	100%	100%	100%	100%	100%
Gaussian Std = 0.1	100%	100%	72%	100%	98%	100%	100%	100%	100%	100%	100%	100%	100%	100%
Gaussian Std = 0.3	100%	100%	100%	100%	100%	100%	100%	100%	100%	100%	100%	100%	100%	100%

## References

[1] Storn R, Price K (1997). Differential evolution—a simple and efficient heuristic for global optimization over continuous spaces. *Journal of Global Optimization*.

[2] Gamperle R, Muller SD, Koumoutsakos P A parameter study for differential evolution.

[3] Zhang J, Sanderson AC An approximate Gaussian model of differential evolution with spherical fitness functions.

[4] Brest J, Greiner S, Bošković B, Mernik M, Zumer V (2006). Self-adapting control parameters in differential evolution: a comparative study on numerical benchmark problems. *IEEE Transactions on Evolutionary Computation*.

[5] Eiben ÁE, Hinterding R, Michalewicz Z (1999). Parameter control in evolutionary algorithms. *IEEE Transactions on Evolutionary Computation*.

[6] Eiben AE, Smith JE (2003). *Introduction to Evolutionary Computing*.

[7] Qin AK, Suganthan PN Self-adaptive differential evolution algorithm for numerical optimization.

[8] Brest J, Boškovć B, Greiner S, Žumer V, Maučec MS (2007). Performance comparison of self-adaptive and adaptive differential evolution algorithms. *Soft Computing*.

[9] Ali M, Pant M (2011). Improving the performance of differential evolution algorithm using Cauchy mutation. *Soft Computing*.

[10] Teo J (2006). Exploring dynamic self-adaptive populations in differential evolution. *Soft Computing*.

[11] Abbass HA The self-adaptive Pareto differential evolution algorithm.

[12] Zhang J, Sanderson AC (2009). JADE: adaptive differential evolution with optional external archive. *IEEE Transactions on Evolutionary Computation*.

[13] Zhang J, Sanderson AC (2009). *Adaptive Differential Evolution: A Robust Approach to Multimodal Problem Optimization*.

[14] Yao X, Liu Y, Lin G (1999). Evolutionary programming made faster. *IEEE Transactions on Evolutionary Computation*.

[15] Yao X, Liu Y, Liang KH, Lin G (2003). Fast evolutionary algorithms. *Advances in Evolutionary Computing*.

[16] Mezura-Montes E, Velázquez-Reyes J, Coello Coello CA A comparative study of differential evolution variants for global optimization.

[17] Vesterstrøm J, Thomsen R A comparative study of differential evolution, particle swarm optimization, and evolutionary algorithms on numerical benchmark problems.

[18] Cuevas E, Zaldivar D, Pérez-Cisneros M (2010). A novel multi-threshold segmentation approach based on differential evolution optimization. *Expert Systems with Applications*.

